# Analysis of maturation dynamics and developmental competence of in vitro matured oocytes under time-lapse monitoring

**DOI:** 10.1186/s12958-021-00868-0

**Published:** 2021-12-10

**Authors:** Qiyu Yang, Lixia Zhu, Meng Wang, Bo Huang, Zhou Li, Juan Hu, Qingsong Xi, Jing Liu, Lei Jin

**Affiliations:** Reproductive Medicine Center, Tongji Hospital, Tongji Medical College, Huazhong University of Science and Technology, No.1095, Jiefang Road, Wuhan, 430030 China

**Keywords:** In vitro maturation, Oocyte maturation dynamics, Time-lapse monitoring, Progesterone, Developmental competence

## Abstract

**Background:**

To improve the developmental competence of in vitro cultured oocytes, extensive literature focused on maturation rate improvement with different additives in culture medium, while studies investigating the maturation dynamics of oocytes during in vitro maturation (IVM) and the influencing factors on oocyte viability are scarce.

**Methods:**

The study involved a retrospective observation by time-lapse monitoring of the IVM process of 157 donated GV oocytes from 59 infertile couples receiving ICSI in 2019, in Tongji Hospital, Wuhan, China. The GV oocytes derived from controlled ovarian hyperstimulation (COH) cycles underwent rescue IVM (R-IVM), and the maturation dynamics, including GVBD time (GV-MI), time from GVBD to maturation (MI-MII), maturation time (GV-MII), and MII arrest duration (MII-ICSI), were recorded by time-lapse monitoring. The matured oocytes were inseminated at different MII arrest points and subsequent embryo developments were assessed. The effects of baseline clinical characteristics, oocyte diameters, and maturation dynamics on the developmental competence of the oocytes were also analyzed.

**Results:**

Totally, 157 GV oocytes were collected. GVBD happened in 111 oocytes, with a median GV-MI duration of 3.7 h. The median MI-MII duration was 15.6 h and the median GV-MII duration was 19.5 h. The maturation rate reached 56.7% at 24 h and 66.9% at 48 h, and the clinical factors, including patient age, FSH level, AMH level, ovarian stimulation protocol, and serum estradiol and progesterone levels on hCG trigger day, showed no effects on the 24-h maturation rate. The normal fertilization rate of oocytes resuming meiosis within 8 h and matured within 24 h was significantly higher than that of oocytes resuming meiosis after 8 h and matured after 24 h. Furthermore, among those oocytes matured within 24 h, the high-quality embryo formation rate of oocytes resuming meiosis within 4.5 h and matured within 19 h was significantly higher. All stated time was measured from the start point of IVM. Additionally, for oocytes from patients with serum progesterone levels less than 1 ng/ml on hCG trigger day, the high-quality embryo formation rate was significantly increased.

**Conclusion:**

R-IVM technology could increase the available embryos for patients in routine COH cycles, but excessive culture beyond 24 h is not recommended. GV-MI duration of the oocyte, recorded by time-lapse system, and serum progesterone levels of patients on hCG trigger day can significantly affect the developmental potential of the IVM oocytes.

## Introduction

In vitro maturation (IVM) of oocytes is a rapidly developing technique in the past three decades, which could be broadly divided into two categories based on the different sources of immature oocytes [1]. The classical IVM implies that the immature oocytes are obtained in natural cycles without any hormone treatments or with minimal stimulation and cultured to maturation in vitro, which is applicable for the patients with polycystic ovary syndrome (PCOS) during assisted reproductive technology (ART), to reduce the risk of ovarian hyperstimulation syndrome (OHSS), and also for fertility preservation of patients with cancer, especially the ones with contraindications to hormone use. Another type of IVM implies the in vitro culture of oocytes that failed to mature in vivo during conventional ovarian stimulation cycles, aiming to increase the available embryo rate in ART and thus improve the success rate, known as rescue IVM (R-IVM) [2, 3].

For poor-prognosis women, the R-IVM has been shown to significantly increase total embryo yields, decrease overall cancellation rates, and support the establishment of a healthy pregnancy [2, 4, 5]. However, studies on IVM in stimulated cycles as a rescue method are rather limited, with a consensus that embryos derived from IVM oocytes have lower developmental competence than sibling embryos derived from oocytes matured in vivo [3, 6, 7], therefore the culture system needs to be improved before generalized clinically. Comparing to the large number of studies focusing on maturation rate improvement with different additives in culture medium [8], studies investigating the maturation dynamics of oocytes during IVM and the influencing factors on oocyte viability are scarce.

Nuclear maturation of oocytes generally contains two stages: GV-MI stage, indicating the resumption of meiosis marked by germinal vesicle breakdown (GVBD), and MI-MII stage, indicating the nuclear maturation marked by extrusion of the first polar body (PB1). With the introduction of the time-lapse monitoring system, which has been mostly applied to the evaluation and selection of embryos [9–11], the dynamics of the nuclear maturation of oocytes during the IVM process could be recorded and assessed. Escrich et al. showed that time-lapse incubators supported up to 70% of germinal vesicle (GV) oocytes to mature in the first 24 h of IVM, and normal activation response of oocytes depended on the duration of nuclear maturation rather than MII arrest duration [12]. However, factors affecting the dynamics of oocytes IVM and subsequent embryo developmental competence were not clear.

In the current study, the dynamics of oocytes maturation during R-IVM following routine ovarian stimulation cycles and subsequent embryo developments were recorded with time-lapse monitoring, and the clinical factors and dynamical parameters affecting oocytes developmental potential were comprehensively analyzed, to reveal the clinical value of these immature oocytes and find the possible intervention target, so as to improve the utilization rate of immature oocytes and the chance of achieving live births.

## Materials and methods

### Study population

Couples undergoing intracytoplasmic sperm injection (ICSI) due to male-factor infertility during the routine ovulation induction cycle from January to December 2019 in our center were reviewed. The ICSI cycles, in which immature GV oocytes were available after denudation, were included. The patients with morphologically abnormal oocytes, normal sperm morphology rate less than 2%, normal fertilization rate of MII oocytes less than 25%, patients undergoing testicular sperm aspiration (TESA) or percutaneous/microsurgical epididymal sperm aspiration (PESA/MESA), and patients in preimplantation genetic testing (PGT) cycles were excluded.

The original study was approved by the Ethical Committee of Tongji Hospital, Tongji Medicine College, Huazhong University of Science and Technology (#[2019] S964). Each of the patients had given written informed consent before the cycle start for the donation of their immature oocytes and sperms for research use.

### Oocytes collection and in vitro maturation

Controlled ovarian hyperstimulation (COH) was processed as previously described [13, 14]. Briefly, pituitary suppression was achieved by injection of GnRH agonist (Triptorelin acetate, Decapeptyl, Ferring) starting in the mid-luteal phase of the previous cycle or GnRH antagonist (Cetrotide, Merck-Serono) starting with the existence of follicles measuring 13–14 mm in diameter. The dosage and duration of recombinant follicle stimulating hormone (FSH) (Gonal-F, Merck-Serono) were adjusted based on individual ovarian response. When two to three leading follicles reached a mean diameter of 18 mm, IM injection of recombinant human chorionic gonadotropin (hCG) (Ovidrel; Merck-Serono) was performed and cumulus-oocyte complexes (COCs) were retrieved by guided transvaginal ultrasound 36–38 h after hCG trigger. The COCs were cultured for 2–3 h in IVF medium (Vitrolife, Sweden) under standard culture conditions (37 °C and 6% CO_2_ in air), after which cumulus cells were removed by 80 IU hyaluronidase (Vitrolife, Sweden) and mechanical stripping to assess the maturity of oocytes.

After denudation, the GV oocytes were collected and cultured in G1-plus medium (Vitrolife, Sweden) in an Embryo Scope (Vitrolife, Sweden) incubator (37 °C, 6% CO_2_ and 5% O_2_ in air) equipped with a time-lapse monitoring system. Images were captured every 10 min. The time when GV oocytes were put into the time-lapse system was set as the starting point, and the dynamical parameters of oocyte nuclear maturation were recorded, including the time of GVBD (GV-MI), time from GVBD to PB1 extrusion (MI-MII), and time of maturation (GV-MII), as well as morphological parameters such as the diameter of oocytes and diameter of GV. ICSI was subsequently performed on mature oocytes using the sperm of the husband of the corresponding patient, and the MII arrest duration before ICSI (MII-ICSI) was also recorded.

### Embryos culture and evaluation following ICSI

After ICSI, oocytes were continuously cultured in G1-plus medium (Vitrolife, Sweden) under time-lapse monitoring. A fertilization check was performed 16–18 h after insemination and the presence of two pronuclei (2PN) was regarded as normal fertilization. The fertilized zygotes were cultured to the cleavage stage until day 3, followed by 2 or 3 days of culture in the G2-plus medium (Vitrolife, Sweden) to the blastocyst stage. Embryo development was assessed on day 2 and day 3 based on the number of blastomeres, the degree of fragmentation, and the blastomere symmetry. High-quality embryos were defined as normally fertilized oocytes with no less than six blastomeres, fragmentation less than 20%, and symmetrical blastomeres on day 3. Blastocysts were graded using a system proposed by Gardner [15]. A blastocyst with >3BB grade on day 5 or > 4BB on day 6 was considered to be of high quality.

### Statistical analyses

**Fig. 1:**
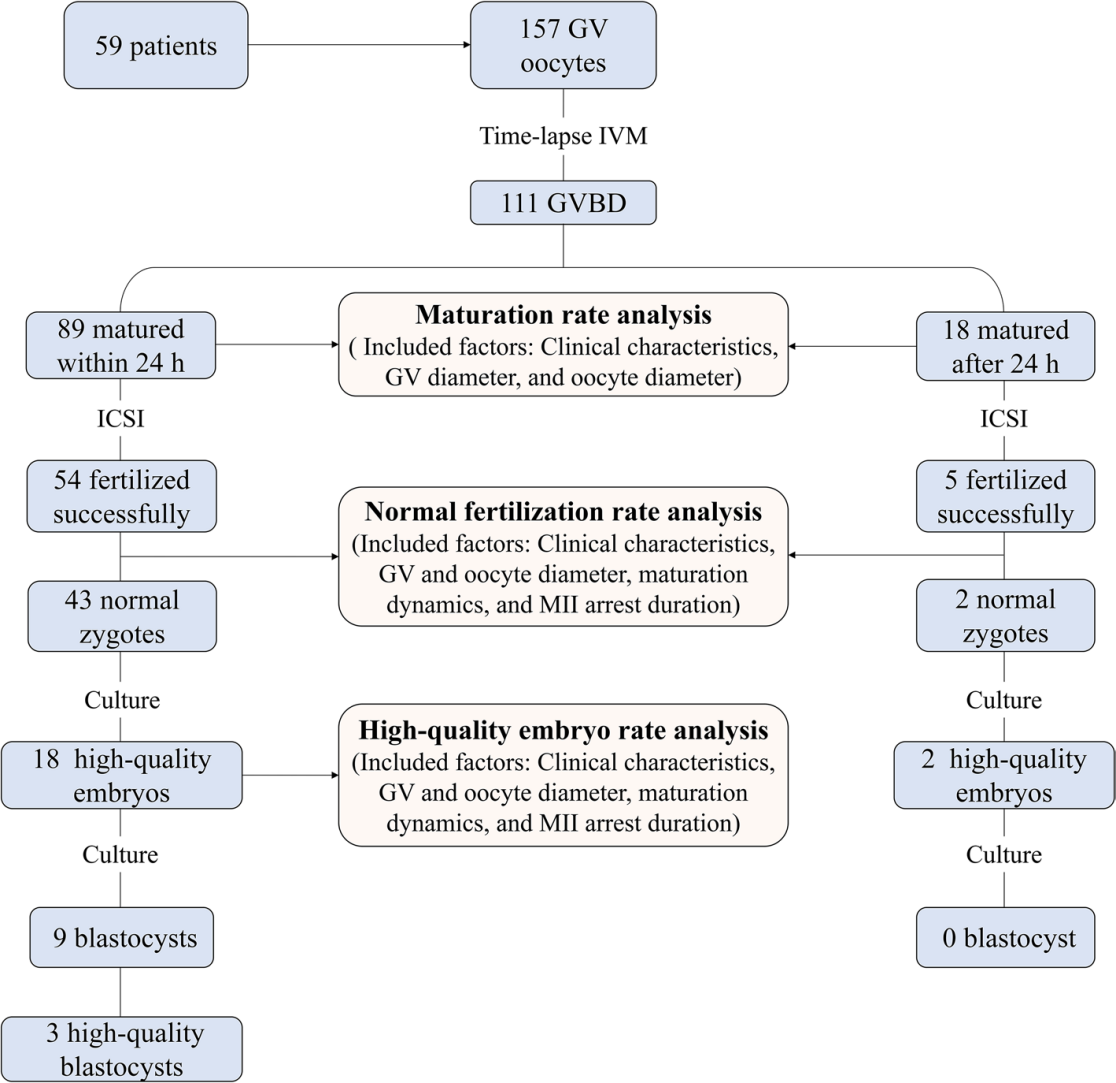
The overall flowchart of data collection and analyses. Baseline characteristics, morphological parameters, and maturation dynamics of the oocytes undergoing R-IVM were comprehensively documented and their effects on 24-h maturation rate, normal fertilization rate, and high-quality embryo formation rate were assessed

Continuous data were expressed as mean ± standard deviation if normally distributed, otherwise as median (interquartile range [IQR]). Categorical data were presented as the number of cases and frequency (percentage). As presented in the flowchart in Fig. 1, clinical factors and dynamical parameters were included as influencing factors of oocyte nuclear maturation rate, normal fertilization rate, and high-quality embryo rate. Oocytes were grouped into different percentiles in involved variables and analyses were performed by Chi-square test. Differences were considered statistically significant when the *P* value was less than 0.05. All statistical analyses were performed using SPSS 22.0 (IBM, USA).

## Results

A total of 157 GV oocytes were collected in 59 ICSI cycles from 59 patients, with a mean age of 31.8 years and a mean body mass index (BMI) of 21.1 kg/m^2^. Most of the patients (69.5%) were diagnosed as primary infertility and 30.5% as secondary infertility, with a median infertility duration of 2.3 years. Other characteristics, including ovarian reserve, stimulation protocols, ovarian response, and matured oocytes development, were presented in Table 1. After in vitro culture, 52 (88.1%) patients obtained 107 matured oocytes and the mean number of successfully rescued oocytes per patient was 1.8 (ranges from 0 to 7). The maturation rate among all oocytes reached 56.7% at 24 h of IVM and 66.9% at 48 h.

**Table 1:** Baseline characteristics of fifty-nine involved female patients

Patient Characteristics (*N* = 59)	Values
Age, years	31.8 ± 0.6
BMI, kg/m^2^	21.1 ± 0.3
Infertility type, n (%)	
Primary	41 (69.5%)
Secondary	18 (30.5%)
Infertility duration, years	2.3 (2–6)
Basal serum FSH level, mIU/ml	6.8 (5.9–7.8)
Antral follicle count	14.9 ± 0.9
Serum AMH level, ng/ml	4.7 (2.9–8.3)
Ovarian stimulation protocol, n (%)	
GnRH agonist protocol	32 (54.2%)
GnRH antagonist protocol	27 (45.8%)
Ovarian response	
Serum E2 level on hCG trigger day, pg/ml	2567.0 (1541.5–4253.0)
Serum P level on hCG trigger day, ng/ml	0.9 (0.6–1.1)
Oocytes obtained, n	14 (8.5–21.0)
Mature oocytes obtained, n	10 (5–17)
Maturation rate at retrieval	68.4%
Normal fertilization rate	73.4%
Available embryo formation rate	68.0%
OHSS rate	8.5%

*BMI* body mass index, *FSH* follicle stimulating hormone, *AMH* anti-Müllerian hormone*; E2* estradiol, *P* progesterone, *OHSS* Ovarian hyperstimulation syndrome

**Fig. 2:**
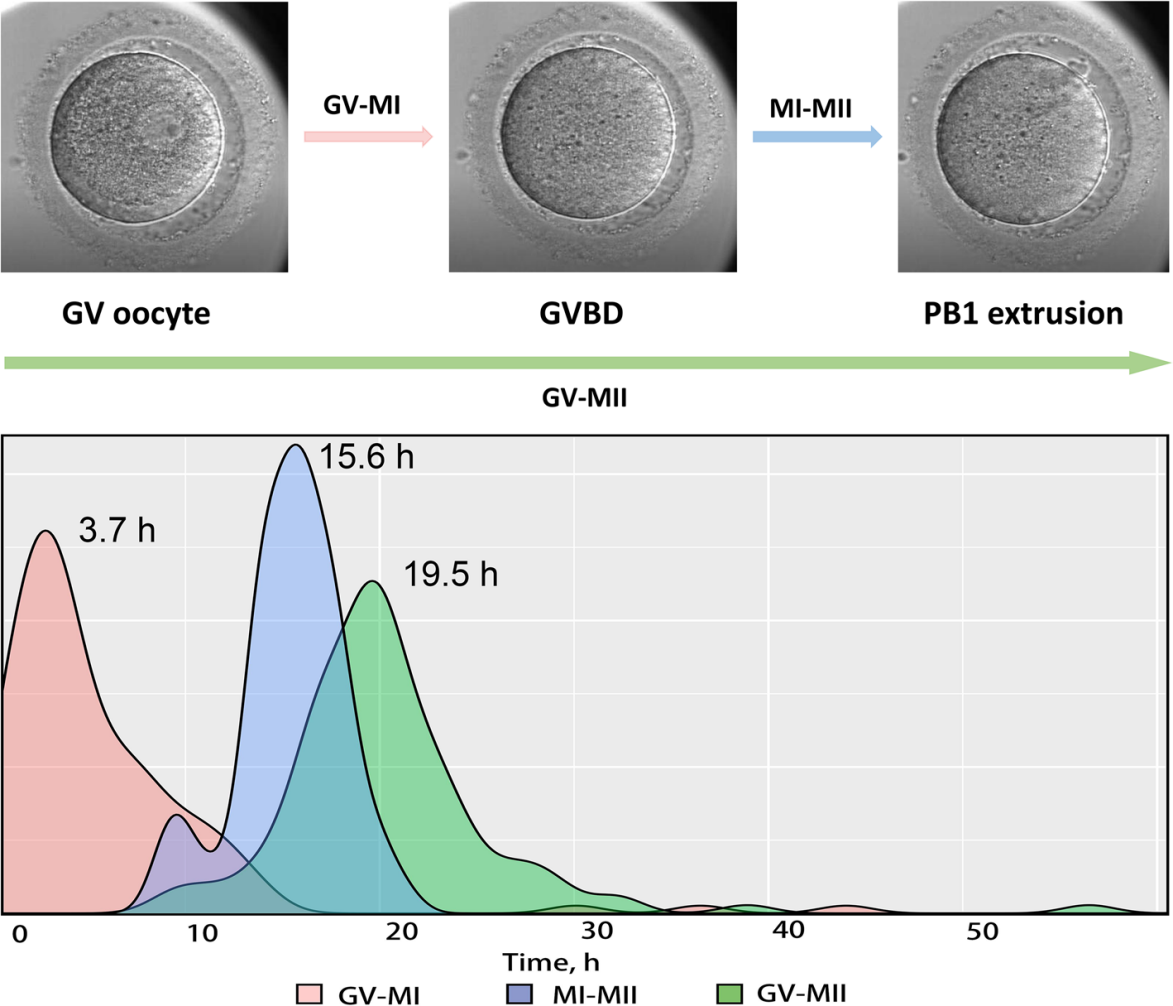
The time distribution of each stage in all oocytes resuming meiosis**.** The duration of GV-MI (the red area) varied between 0.3 h and 44 h, with a dispersion coefficient of 0.95. The duration of MI-MII (the blue area) ranged from 8.6 h to 21.5 h, with a dispersion coefficient of 0.13. The duration of maturation (the green area) ranged from 9.1 h to 64 h, with a dispersion coefficient of 0.20. The numbers marked on the image were the median duration of GVBD, MI-MII, and maturation

The data from time-lapse monitoring system showed that the median diameter of the 157 GV oocytes was 110 μm (IQR: 108–112 μm), with a median GV diameter of 31 μm (29 - 31 μm). During IVM, GVBD happened in 111 oocytes, with a median GV-MI time of 3.7 h (2.2–7.5 h). The median MI-MII time was 15.6 h (14.0–17.3 h) and the median GV-MII time was 19.5 h (17.3–22.4 h). The time distribution of each period in all matured oocytes was shown in Fig. 2.

The IVM oocytes were subsequently divided into two groups in terms of maturation time (within 24 h or after 24 h) to evaluate their fertilization rate and embryo development, which were shown respectively in the flow chart in Fig. 1. Among the 89 oocytes matured within 24 h, 54 oocytes were successfully fertilized after ICSI and 43 normal zygotes were obtained, which subsequently formed 18 high-quality embryos in day 3 and 9 blastocysts in day 5. Of the 18 oocytes matured after 24 h, merely 2 oocytes were normally fertilized and 2 high-quality embryos obtained, yet no blastocyst formed. It seemed that the oocytes matured after 24 h presented lower developmental potential and availability, with decreased fertilization rate and high-quality embryo formation rate, comparing with those matured within 24 h.

We further analyzed factors influencing the 24-h maturation rate, fertilization rate, and high-quality embryo formation rate of IVM human oocytes. As presented in Table 2, the potential factors, including patient age, basal FSH level, anti-Müllerian hormone (AMH) level, ovarian stimulation protocol, serum estradiol (E2) level and serum progesterone (P) level on hCG trigger day, GV diameter, and oocyte diameter, showed no significant effects on the 24-h maturation rate of human GV oocytes. ICSI procedures were performed in 104 matured oocytes (with 3 oocytes discarded due to dysmorphosis) following the extrusion of PB1, and 45 normal zygotes were obtained totally. The analysis of influencing factors (shown in Table 3) indicated that the normal fertilization rate of oocytes matured within 24 h of IVM was significantly higher than that of oocytes matured after 24 h (48.3% vs. 13.3%, *P* = 0.011). The effect seemed to be more closely related to the GV-MI duration rather than the MI-MII time, as the normal fertilization rate of oocytes resuming meiosis within 8 h significantly exceeded that of oocytes with GVBD time over 8 h (49.4% vs. 11.8%, *P* = 0.004). No significant difference was observed in the oocytes from patients with varying age, basal FSH level, AMH level, E2, and P level on hCG trigger day, nor in the oocytes with different diameters. The MII-ICSI duration showed no significant effect on the normal fertilization rate of IVM oocytes.

**Table 2:** Analysis of factors affecting 24-h maturation rate of 157 GV oocytes during R-IVM

	Maturation rate in 24 h, %	Chi square	*P* value	OR	95% CI
Age of patients involved, years		0.006	0.936	0.974	0.517–1.837
< 33 (*n* = 86)	57.0				
≥ 33 (*n* = 71)	56.3				
FSH, mIU/ml		0.222	0.638	1.174	0.602–2.288
< 7 (*n* = 103)	55.3				
≥ 7 (*n* = 54)	59.3				
AMH, ng/ml		0.044	0.835	1.080	0.524–2.226
< 4 (*n* = 41)	55.0				
≥ 4 (*n* = 116)	56.9				
Ovarian stimulation protocol		0.037	0.848	1.064	0.561–2.019
GnRH agonist protocol (*n* = 91)	56.0				
GnRH antagonist protocol (*n* = 66)	57.6				
E2 on hCG day, pg/ml		0.330	0.566	0.831	0.442–1.563
< 3000 (*n* = 78)	59.0				
≥ 3000 (*n* = 79)	54.4				
P on hCG day, ng/ml		0.087	0.786	1.100	0.585–2.070
< 1 (*n* = 81)	55.6				
≥ 1 (*n* = 76)	57.9				
GV diameter, μm		0.006	0.938	0.973	0.492–1.925
< 30 (*n* = 49)	57.1				
≥ 30 (*n* = 108)	56.5				
Oocyte diameter, μm		0.011	0.917	1.036	0.537–1.996
< 110 (*n* = 57)	56.1				
≥ 110 (*n* = 100)	57.0				

*FSH* follicle stimulating hormone*, AMH* anti-Müllerian hormone*, E2* estradiol*, P* progesterone*, GV* germinal vesicle

**Table 3:** Analysis of factors influencing normal fertilization rate of 104 matured oocytes following ICSI

	Normal fertilization rate, %	Chi square	*P* value	OR	95% CI
Patient age, years		2.239	0.135	0.548	0.249–1.208
< 33 (*n* = 56)	50.0				
≥ 33 (*n* = 48)	35.4				
FSH, mIU/ml		0.351	0.553	0.783	0.348–1.761
< 7 (*n* = 66)	45.5				
≥ 7 (*n* = 38)	39.5				
AMH, ng/ml		0.027	0.087	1.078	0.439–2.649
< 4 (*n* = 27)	42.3				
≥ 4 (*n* = 77)	44.2				
E2 on hCG day, pg/ml		1.777	0.182	1.700	0.777–3.718
< 3000 (*n* = 54)	37.0				
≥ 3000 (*n* = 50)	50.0				
P on hCG day, ng/ml		0.001	0.979	0.990	0.455–2.150
< 1 (*n* = 53)	43.4				
≥ 1 (*n* = 51)	43.1				
GV diameter, μm		1.829	0.176	0.561	0.242–1.301
< 30 (*n* = 32)	53.1				
≥ 30 (*n* = 72)	38.9				
Oocyte diameter, μm		1.105	0.293	0.650	0.291–1.454
< 110 (*n* = 38)	50.0				
≥ 110 (*n* = 66)	39.4				
GV-MI, h		8.217	**0.004**	0.136	0.029–0.633
< 8 (*n* = 87)	49.4				
≥ 8 (*n* = 17)	11.8				
MI-MII, h		0.863	0.353	0.690	0.315–1.512
< 16 (*n* = 57)	47.4				
≥ 16 (*n* = 47)	38.3				
GV-MII, h		6.399	**0.011**	0.165	0.035–0.772
< 24 (*n* = 89)	48.3				
≥ 24 (*n* = 15)	13.3				
MII-ICSI, h		0.410	0.522	0.769	0.344–1.719
< 6 (*n* = 38)	47.4				
≥ 6 (*n* = 66)	40.9				

*FSH* follicle stimulating hormone*, AMH* anti-Müllerian hormone*, E2* estradiol*, P* progesterone*, GV* germinal vesicle

The factors affecting high-quality embryo formation rate of 54 zygotes derived from oocytes matured within 24 h were further investigated (shown in Table 4). The results suggested that the high-quality embryo formation rate of oocytes matured within 19 h was significantly higher than that of oocytes matured after 19 h (52.2% vs. 19.4%, *P* = 0.011). Similarly, a GV-MI duration less than 4.5 h also had a positive effect on high-quality embryo formation rate (42.1% vs. 12.5%, *P* = 0.035). In addition, the serum P levels on hCG trigger day significantly affected the high-quality embryo formation rate. For oocytes from patients with serum P levels less than 1 ng/ml, the high-quality embryo formation rate was significantly higher (48.0% vs. 20.7%, *P* = 0.034). Other factors showed no effects on the high-quality embryo formation rate.

**Table 4:** Analysis of factors influencing the high-quality embryo formation rate of 54 zygotes derived from oocytes matured within 24 h

	High-quality embryo formation rate, %	Chi square	*P* value	OR	95% CI
Patient age, years		0.162	0.687	1.273	0.393–4.117
< 33 (*n* = 35)	31.4				
≥ 33 (*n* = 19)	36.8				
FSH, mIU/ml		1.015	0.314	1.818	0.565–5.854
< 7 (*n* = 35)	28.6				
≥ 7 (*n* = 19)	42.1				
AMH, ng/ml		3.726	0.071	0.258	0.062–1.078
< 4 (*n* = 11)	60.0				
≥ 4 (*n* = 43)	27.9				
E2 on hCG day, pg/ml		0.149	0.700	0.800	0.257–2.486
< 3000 (*n* = 25)	36.0				
≥ 3000 (*n* = 29)	31.6				
P on hCG day, ng/ml		4.506	**0.034**	0.283	0.086–0.932
< 1 (*n* = 25)	48.0				
≥ 1 (*n* = 29)	20.7				
GV diameter, μm		3.375	0.066	0.333	0.101–1.099
< 30 (*n* = 18)	50.0				
≥ 30 (*n* = 36)	25.0				
Oocyte diameter, μm		0.375	0.540	0.691	0.212–2.258
< 110 (*n* = 18)	38.9				
≥ 110 (*n* = 36)	30.6				
GV-MI, h		4.441	**0.035**	0.196	0.039–0.988
< 4.5 (*n* = 38)	42.1				
≥ 4.5 (*n* = 16)	12.5				
MI-MII, h		0.993	0.319	0.538	0.158–1.835
< 16 (*n* = 34)	38.2				
≥ 16 (*n* = 20)	25.0				
GV-MII, h		6.400	**0.011**	0.220	0.066–0.738
< 19 (*n* = 23)	52.2				
≥ 19 (*n* = 31)	19.4				
MII-ICSI, h		1.403	0.236	2.080	0.612–7.067
< 6 (*n* = 21)	23.8				
≥ 6 (*n* = 33)	39.4				

*FSH* follicle stimulating hormone, *AMH* anti-Müllerian hormone, *E2*,estradiol, *P* progesterone, *GV*s germinal vesicle

## Discussion

In the current study, we recorded the dynamical parameters of R-IVM of human immature oocytes derived from routine COH cycles, using a time-lapse monitoring culture system, and investigated the effects of different clinical characteristics and maturation dynamics on the IVM oocytes and subsequent developmental potential of embryos after fertilization.

Among the total 157 GV oocytes, 68.2% were matured during the R-IVM, providing extra matured oocytes for 52 (88.1%) patients. In addition, no significant effects of clinical factors on the maturation rate within 24 h were observed. Taken together, immature oocytes collected from routine COH cycles still have a great potential to reach maturation by in vitro culture, regardless of the clinical characteristics, ovarian stimulation protocol, and oocyte diameter, confirming the necessity and prospect of the R-IVM [3, 16–21].

Moreover, 56.7% of oocytes matured within 24 h, which led to a higher normal fertilization rate comparing with those matured in 24–48 h. This result was consistent with previous research, which showed that early maturing oocytes (18.4 ± 2.7 h) reached higher normal activation rates comparing to late maturing oocytes (26.3 ± 3.8 h) [12]. An earlier study has also demonstrated that the rates of cleavage and blastocyst development of oocytes matured on day 2 after IVM were significantly lower than those matured on day 1 [22]. Both the current and previous studies proved the utilization value of oocytes matured within 24 h of R-IVM. Nevertheless, for the few oocytes matured in 24 h - 48 h of R-IVM, the normal fertilization rate, high-quality embryo formation rate, and blastocyst formation rate were dramatically decreased. Given the low utilization value of oocytes matured in 24–48 h and the time consumption of embryologists by prolonged culture, extended IVM beyond 24 h is not recommended clinically in the current IVM culture condition.

Similar to the 24-h maturation rate, the normal fertilization rate of oocytes after R-IVM was not influenced by the patients’ clinical characteristics and hormone levels. Instead, both GV-MI time and GV-MII time significantly affected the normal fertilization rate of the inseminated mature oocytes, exhibiting a higher 2PN rate in oocytes resuming meiosis within 8 h and matured within 24 h, while no significant differences were identified among oocytes with different MI-MII time or different MII-ICSI time. Additionally, the maturation dynamics of every oocyte recorded by the time-lapse monitoring system showed greater variability in the GV-MI time than in the MI-MII time, which was consistent with previous research, showing that the duration of GV-MI stage differed between the early maturing oocytes and late maturing oocytes, while MI arrest duration was relatively constant [12].

The importance of GV-MI duration was also highlighted when evaluating the high-quality embryo formation rate of the zygotes derived from oocytes matured within 24 h, and analyzing its influencing factors. The results showed that GV-MI time less than 4.5 h and GV-MII time less than 19 h were both positive factors for improving high-quality embryo formation rate. A similar effect of GVBD timing on the subsequent developmental potential of IVM oocytes was also observed in mice and buffaloes [23, 24], yet with an unclear mechanism.

Different from many recent studies focusing on prolonging meiotic arrest by the pre-IVM system to improve oocyte development [25, 26], our study reveals the association between the nuclear maturation process of a single oocyte and its developmental potential, in a standard IVM system without adding any special GVBD blockers. In such a situation, oocytes tend to resume meiosis spontaneously after releasing from their follicles and denuding the cumulus cells, based on the fact that inhibitory follicular environment and gap junction communication between oocyte and somatic cells are both crucial to maintain meiotic arrest [27–29]. Therefore, unprovoked prolonging of GVBD time may indicate an abnormally slow decrease of intraoocyte cyclic adenosine monophosphate (cAMP) level and consequent inactivation of maturation promoting factor (MPF), which would not only disrupt meiotic resumption but also influence successful fertilization, cleavage, and subsequent embryonic mitosis [30–32]. Besides, according to the above-mentioned study regarding time-lapse monitoring IVM, the oocyte developmental incompetence also might be attributed to aged spindles, tripolar or multipolar spindle poles, and chromosome disorganization [12].

Taken together, the in vitro developmental potential of oocytes was closely related to their nuclear maturation dynamical parameters, and the GV-MII duration rather than the MII arrest duration was more critical. Additionally, the maturation time seemingly depended on the GV-MI duration instead of the MI-MII duration. So we believed that during R-IVM, GVBD time could be used as an important indicator to predict oocyte developmental potential, and also as an intervention target for future studies to obtain more high-quality embryos, thereby improving pregnancy and live birth rate.

Apart from the impact of dynamical parameters, a significant effect of serum P levels on hCG trigger day on the high-quality embryo formation rate was identified, indicating that elevated P level on hCG trigger day was unfavorable for improving embryo quality. Our previous study involving 4236 IVF cycles revealed that elevated serum P level on hCG trigger day was associated with a decreased top-quality embryo rate [33], which was consistent with the results of other IVF centers [34–36]. These previous studies focused on the effect of elevated serum P level on the quality of the embryos derived from in vivo matured oocytes, and to our knowledge, the current study is the first to show that high serum P levels after ovulation induction also have a negative impact on the quality of embryos that stem from R-IVM oocytes. One possible explanation proposed by earlier studies suggested that the effect of elevated serum P levels on oocyte or embryo quality may be attributed to the changed follicular environment or dynamics and the altered expression of genes involving steroidogenesis, translation, and cell apoptosis [37, 38].

According to the previous study, some strategies could be taken to deal with elevated P levels, including the individualization of ovarian stimulation protocol and dosage, corticosteroid administration, cycle segmentation with freeze-all-policy, and avoidance of prolonged stimulation [39]. However, whether freezing all embryos is the perfect strategy to rescue the detrimental impacts of elevated P levels is worth pondering, especially when increasing evidence demonstrate the impact of high P levels on oocyte or embryo qualities and the impairment risk from the process of freezing. A better suggested strategy in dealing with elevated P levels might be an earlier oocyte retrieval, which, however, would potentially increase the number of immature oocytes and reduce the available embryos. In this situation, the necessity of developing and improving IVM technology is highlighted, based on which an earlier oocyte retrieval before the P levels reach a certain threshold could be performed, as the obtained immature oocytes could be safely cultured in vitro and generate available embryos. Through the combination of advanced oocyte retrieval and IVM, the detrimental effects of elevated P levels on the endometrium and oocyte qualities might be avoided simultaneously.

As mentioned before, IVM is not only a supplement to IVF-ET technique to increase the available embryo rate, but also an effective method to prevent OHSS by reducing the hormone dosage and shortening the stimulation duration during COH [1]. For patients with PCOS or with high ovarian response, the acquisition of a large number of immature oocytes could provide a considerable reserve after IVM, both for the patients themselves and the oocyte donation program. Additionally, our previous research has proved by aneuploidy analyses that embryos with normal karyotype could be obtained from the IVM oocytes, indicating the prospect of PGT-A in ensuring the safety of IVM oocytes [40]. It is promising that combined application of IVM and PGT-A could provide extra normal embryos, not only for the owner of the oocytes, but also for women undergoing oocyte donation cycles.

In the present study, the dynamics of oocyte maturation were recorded with the time-lapse monitoring system, similar to the previous research [12]. More importantly, we initially analyzed the effects of both the clinical characteristics and the dynamical parameters on the developmental potential of R-IVM oocytes. The results indicated that the oocytes failing to mature at the moment of retrieval during routine COH cycle can still reach maturation by a rescue in vitro culture rather than being discarded. Among them, oocytes matured within 24 h, especially within 19 h, have a high developmental potential after fertilization, while serum P levels of patients on the hCG trigger day have a negative impact on the subsequent formation of high-quality embryos. Clinically, the dynamical parameters of oocytes could be obtained in real time via the time-lapse monitoring system. And through our analyses of both clinical and dynamical data, we found the indicators to predict the IVM outcome of each oocyte (e.g. serum P level and GV-MI duration) and the key developmental process (e.g. GV-MI stage) to intervene, so as to improve the oocyte quality, and increase the available embryo rate, and finally benefit the patients.

Given that R-IVM is still an experimental technique with controversies [41, 42], embryo transfer was not performed and implantation rates and pregnancy outcomes could not be assessed, which is the main limitation of the current study. Comparing with the classical IVM, during which the COCs were mostly used instead of the denuded oocytes, the developmental competence of oocytes in our study was relatively lower [26, 43]. There are two possible explanations: on the one hand, the GV oocytes used in R-IVM are those having failed to mature in vivo after COH, with intrinsically poor developmental potential, so the proportion that can mature in vitro is limited; on the other hand, the absence of cumulus cells may also affect the maturation and subsequent development of oocytes [44–46]. Besides, the sample size of our study was rather limited, due to its single-center nature and the strict inclusive criteria involving both the wife and husband.

## Conclusions

In conclusion, R-IVM technology could increase the available embryos for patients in routine COH cycles, but excessive culture beyond 24 h is not recommended. GV-MI duration of the oocyte, recorded by time-lapse system, and serum progesterone levels of patients on hCG trigger day can be used as indicators to predict the developmental potential of individual oocytes and as potential regulatory targets in future studies to improve IVM outcomes.

## Data Availability

All data generated or analysed during this study are included in this published article.

## Abbreviations

IVM: In vitro maturation
PCOS: Polycystic ovary syndrome
ART: Assisted reproductive technology
OHSS: Ovarian hyperstimulation syndrome
GVBD: Germinal vesicle breakdown
PB1: First polar body
ICSI: Intracytoplasmic sperm injection
COH: Controlled ovarian hyperstimulation
GnRH: Gonadotropin-releasing hormone
FSH: Follicle-stimulating hormone
hCG: Human chorionic gonadotropin
COCs: Cumulus-oocyte complexes
2PN: Two pronuclei
BMI: Body mass index
AMH: Anti-Müllerian hormone
E2: Estradiol
P: Progesterone
